# CAF-1 Subunits Levels Suggest Combined Treatments with PARP-Inhibitors and Ionizing Radiation in Advanced HNSCC

**DOI:** 10.3390/cancers11101582

**Published:** 2019-10-17

**Authors:** Francesco Morra, Francesco Merolla, Ida Picardi, Daniela Russo, Gennaro Ilardi, Silvia Varricchio, Federica Liotti, Roberto Pacelli, Luca Palazzo, Massimo Mascolo, Angela Celetti, Stefania Staibano

**Affiliations:** 1 Institute for the Experimental Endocrinology and Oncology, National Research Council, 80131 Naples, Italy; fmorra85@gmail.com (F.M.); idapicardi93@icloud.com (I.P.); fedeliotti@hotmail.com (F.L.); l.palazzo@ieos.cnr.it (L.P.); 2 Department of Advanced Biomedical Sciences, University “Federico II”, Surgical Pathology Section, 80131 Naples, Italy; francesco.merolla@unimol.it (F.M.); daniela.russo@unina.it (D.R.); gennaro.ilardi@gmail.com (G.I.); silvia.varricchio@gmail.com (S.V.); pacerto@yahoo.com (R.P.); mmascol@gmail.com (M.M.); 3 Department of Medicine and Health Sciences “V. Tiberio”, University of Molise, 86100 Campobasso, Italy

**Keywords:** head and neck squamous cell carcinoma, CAL27, SCC90, p60/p150 CAF-1 subunits, DNA repair, homologous recombination, biomarkers, personalized treatment

## Abstract

Oral (OSCC) and oropharyngeal (OPSCC) squamous cell carcinomas show high morbidity and mortality rates. We aimed to investigate the role of the “Chromatin Assembly Factor-1” (CAF-1) p60 and p150 subunits, involved in DNA repair and replication, in OSCC and OPSCC progression and in response to Poly(ADP-ribose) polymerase (PARP)-inhibitors and exposure to ionizing radiation (IR). We immunostained tissue microarrays (TMAs), including 112 OSCC and 42 OPSCC, with anti-CAF-1/p60 and anti-CAF-1/p150 specific antibodies, correlating their expression with prognosis. Moreover, we assessed the sensitivity to PARP inhibitors and the double-strand breaks repair proficiency by cell viability and HR reporter assays, respectively, in HPV-positive and HPV-negative cell lines upon CAF-1/p60 and CAF-1/p150 depletion. The immunohistochemical analysis revealed a significant prognostic value of both tissue biomarkers combined expression in OSCC but not in OPSCC. In in vitro studies, the p60/150 CAF-1 subunits’ depletion impaired the proficiency of Homologous Recombination DNA damage repair, inducing sensitivity to the PARP-inhibitors, able to sensitize both the cell lines to IR. These results indicate that regardless of the prognostic meaning of p60/p150 tissue expression, the pharmacological depletion of CAF-1 complex’s function, combined to PARP-inhibitors and/or IR treatment, could represent a valid therapeutic strategy for squamous cell carcinomas of head and neck region.

## 1. Introduction

Head and neck cancer (HNC) is the eighth most common malignancy in the world, with *squamous cell carcinoma* (*SCC*) accounting for *more than 90*% [1]. HNSCC encompass tumours of larynx, hypopharynx, paranasal sinuses, oral cavity, and oropharynx. Of all HNSCC, oral cavity SCC (OSCC) and oropharynx SCC (OPSCC) represent more than a half-million new cases per year, with an estimated incidence of 7.0 per 100,000 inhabitants, worldwide. HNSCC are characterized by a high-rate of morbidity and mortality. Furthermore, in most countries, the five-year survival rate is less than 50% and, in the United States, more than 20,000 new cases are estimated to occur in 2019, with more than 10,000 deaths [1,2,3]. Multiple risk factors contribute to the initiation of HNSCC. Tobacco and heavy use of alcoholic beverages, for OSCC, and high-risk Human Papilloma Virus, mainly HR-HPV16 persistent infections, for a significant percentage of OPSCC, are the most important risk-factors, causing a substantial percentage of these tumours in the Western countries. HPV16 accounts for >80% of HPV-positive oropharyngeal SCCs compared with HPV-positive oral SCCs and laryngeal SCCs. Conversely, HPV18 is relatively rare in HPV positive oropharyngeal SCCs compared with other head and neck sites.

While OSCC are commonly observed in males, aged over 50 years, OPSCC occurs according a bi-modal pattern, respectively, in those under 40 and in elderly people [4,5,6], and mainly affect females. OPSCC comprise of HPV-negative (HPV−) and HPV-positive (HPV+) tumours, which represent two distinctive clinicopathological and molecular entities, with a disparate range of survival rates. HPV+ OPSCC are characterized by a significantly slow progression and a high response to chemo- and radiotherapy, while HPV− OPSCC and tobacco smoking/alcohol-related OSCC are intrinsically highly aggressive, and highly chemo- and radio-resistant when in an advanced stage. Currently, there are still no reliable prognostic and predictive biomarkers for these deadly cancers that kill about 50% of patients with metastatic disease. Genotoxic exposure to tobacco carcinogens and consequent adducts formation resulting in DNA damage [7] and genomic instability induced by the unscheduled cell replication of integrated HR-HPVs, as demonstrated in in vitro experiments [8], are thought to represent important mechanisms of carcinogenesis for HPV− and HPV+ HNSCC, respectively. At least five major DNA repair pathways—base excision repair (BER), nucleotide excision repair (NER), mismatch repair (MMR), homologous recombination (HR), and non-homologous and joining (NHEJ)—are active throughout different stages of the cell cycle, allowing the cells to repair the DNA damage in a substrate-dependent manner [9,10,11]. The chromatin assembly factor 1 (CAF-1), a heterotrimeric protein complex formed of three subunits (p48, p60 and p150), plays a key role in the steric organization of DNA and in the assembly of nucleosomes [12]. In particular, while p48 subunit acts on acetylation/deacetylation of histones, p150 appears to be more active in interphase DNA-damage repair process, interacting with PCNA on the damaged DNA, specifically during NER [13,14] and double-strand breaks repair [15], the CAF-1/p60 subunit is more specifically connected to controlling cell replication [16,17,18]. Several reports have recently shown that a deregulated expression of this subunit leads cells to incorrectly replicate DNA, consequently accumulating DNA damage [16,19]. Interestingly, CAF-1/p60 expression levels are significantly correlated with the biological aggressiveness of tumours, metastasizing behaviour and worse prognosis in breast, oral, prostate, laryngeal and salivary gland carcinomas, as well as in skin melanoma [20,21,22,23,24,25], suggesting for this protein a promising role as a new sensible prognostic marker, apparently unrelated to their histogenesis. In recent decades, despite therapeutic advances in the HNSCC treatment, patient survival has not markedly improved and the mortality is still around 40–50%. CAF-1 appears as an interesting candidate to explore in HNSCC. In the present study, we evaluated the expression of CAF-1/p60 and p150 subunits in a tissue microarray (TMA) selected series of OSCC and OPSCC. We related our data to the HPV status of primary OPSCC, evaluated by immunohistochemical expression of p16^INK4a^ protein. We also verified the prognostic value of these tissue biomarkers through follow-up analysis. We used, as models of in vitro study, in vitro cultured cells of OSCC and HPV+ OPSCC, testing the efficiency of repair mechanisms and radiation sensitivity, upon silencing of CAF-1/p60 and p150. 

## 2. Results

### 2.1. Immunohistochemistry Expression of CAF-1/p60 and p150 Subunits in OSCC and OPSCC Tumour Samples

**Figure 1:**
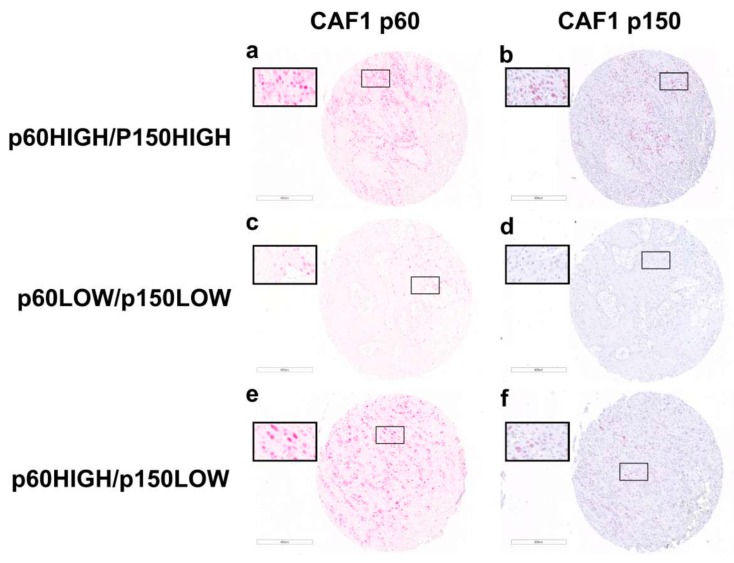
IHC staining of OSCC FFPE tumour samples with anti-CAF-1 p60 and anti-CAF-1 p150 antibodies. The figure shows representative images of anti-CAF-1 p60 and anti-CAF-1 p150 IHC staining intensity in OSCC FFPE tumour samples grouped according to cluster classification as resulted by cluster analysis of immunohistochemistry expression data: (**a**,**b**) p60 HIGH and p150 HIGH staining category, respectively; (**c**,**d**) p60 LOW and p150 LOW staining category, respectively; and (**e**,**f**) p60 HIGH and p150 LOW staining category, respectively. Magnification: for each panel, a 5× image of the entire core is shown and the inset shows the highlighted region.

To investigate the functional role of the CAF-1/p60 and p150 subunits in HNSCC, we evaluated a Tissue microarray (TMA) case study (total number of cases *N* = 154) of non-oropharyngeal (OSCC) (*N* = 112) and oropharyngeal squamous cell carcinomas (OPSCC) (*N* = 42), the latter tested for the presence of the HPV virus (HPV+ OPSCC *N* = 8). All tumour samples were staged according to the 8th AJCC staging manual [26] (Table 1). In these samples, the immunohistochemistry staining for the p60 and p150 subunits of CAF-1 was assessed and scored as “HIGH” and “LOW” as defined in the Section 4 (representative images of staining score categories are shown in Figure 1). The p60/p150 frequency distribution data were further analysed with a classification algorithm allowing stratification of samples in three clusters, homogeneous for tissue expression of p60 and p150 according to the IHC staining. The three clusters were defined as follow: p150 HIGH/p60 HIGH; p150^LOW^/p60^HIGH^; and p150^LOW^/p60^LOW^. The category p150^HIGH^/p60^LOW^ was not revealed since no p60LOW cases were present in the p150^HIGH^ sub-group (Table 1).

To analyse the frequency distribution of CAF-1/p60, CAF-1/p150, and overall survival (OS) variables, we set up a contingency table.

By applying Fisher’s exact test, the frequency distribution of p60 and p150 positivity, crossed by OS, proved to be statistically significant in the whole study population (*p* = 0.022), statistical significance was particularly high in OSCC group (*p* = 0.013) and no significance resulted from OPSCC samples analysis (*p* = 0.485) (Table S1). 

By contingency table analysis, we could observe that, in the whole tested population, p60^HIGH^ score mostly segregates with a poor prognosis, in terms of overall survival, as expected (dead/alive ratio = 1.61 in p60^HIGH^, 0.68 in p60^LOW^). Moreover, the association of p60 with the worst outcome was even stronger in the p150^LOW^ score group (dead/alive ratio = 1.89). The analysis of p60 and p150 frequency distribution revealed the highest dead/alive ratio in the p60^HIGH^/p150^LOW^ population of OSCC samples (45/18 = 2.5), while p60 expression did not correlate with outcome in OPSCC samples (*p* = 0.485). Nevertheless, out of eight HPV+ OPSCC samples, the only one presenting a poor outcome at follow-up belonged to p60^HIGH^/p150^LOW^ subgroup.

**Figure 2:**
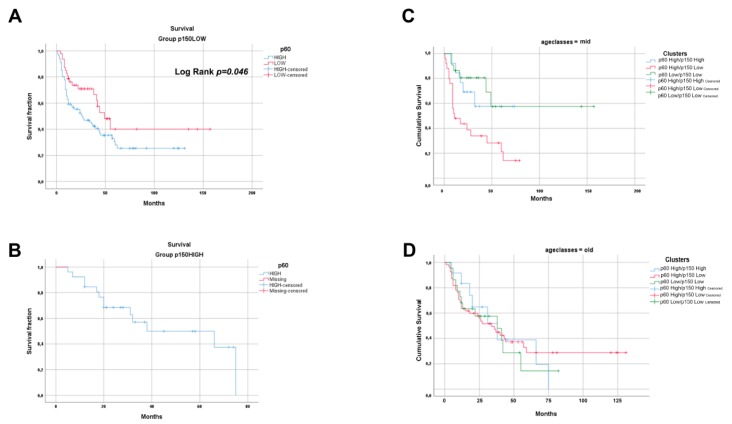
Survival analysis by Kaplan–Meier curves. The picture shows CAF-1 p60 HIGH and CAF-1 p60 LOW survival curves in the study population grouped by CAF-1 p150 staining score. (**A**) CAF-1 p60 HIGH and CAF-1 p60 LOW curves in p150 LOW group. (**B**) Since CAF-1 HIGH category is only associated with CAF-1 p60 HIGH staining score, only this curve is shown. Statistical differences between curves were assessed by log-rank test, where applicable. (**C**,**D**) Kaplan–Meier curves survival analysis of the three clusters stratified by age class were performed (**C**) for age class “41–60” and (**D**) for age class “>60”. Difference between curves was statistically significant in mid-age group (40–60 years old) (Log-Rank test *p* = 0.005).

Survival curves analysis confirmed a statistically significant difference between p60^HIGH^ and p60^LOW^ curves in the p150^LOW^ population, (log-rank test, *p* = 0.0034). A not-significant *p*-value was obtained evaluating the differences in OPSCC group (*p* = 0.477), irrespective of HPV status (Figure 2). 

To further unravel the prognostic potential of p60 and p150 tissue expression in OSCC, we stratified the studied population by the age of patients at diagnosis, grouping the population in “young” (<40 years); “mid” (41–60 years); and “old” (>60 years). The survival data frequency distribution analysis, upon crossing the clusters by the overall survival results and the age groups, gave an extremely significant result in the mid-age population (*p* = 0.002). In the old age population, the distribution was not significant (*p* = 0.910), and the group of young age population was not big enough to allow statistical analysis (*N* = 5) (Table S2).

The tissue overexpression of CAF-1/p60 subunit associates with poor overall survival in OSCC, in the absence or with concomitant low expression of CAF-1/p150 subunit. We did not observe a significant association with the outcome in OPSCC tumour samples.

### 2.2. The Silencing of the CAF-1 Subunits Increases the Sensitivity to Ionizing Radiation in HPV-Negative and HPV-Positive Head and Neck Cancer Cell Lines

The protein “chromatin Assembly Factor-1” (CAF-1) plays a fundamental role in the steric organization of DNA and the assembly of Nucleosomes [12], thanks to its subunits, p60, p48 and p150 that make up the Heterotrimer CAF-1 [13,17,18]. CAF1 functions as “histone chaperone”, and its activity is also required during the DNA damage repair [12,27]. While CAF-1/p150 subunit appears to be more active in interphase DNA damage repair processes, interacting with PCNA on the damaged DNA, during nucleotide excision repair (NER) [10,14] and double-strand breaks repair [15], CAF-1/p60 has been described to be more specifically connected to cell replication. Nevertheless, loss of p60 leads the cells to incorrectly replicate DNA, consequently accumulating DNA damage [16].

In this work, by utilizing in vitro cultured cells of HPV-negative and HPV-positive oral cavity squamous cell carcinoma, we investigated whether the stable silencing of the CAF1 subunits, p60, p150 or p60 and p150 together, could modulate the response to ionizing radiation (IR) therapy.

**Figure 3:**
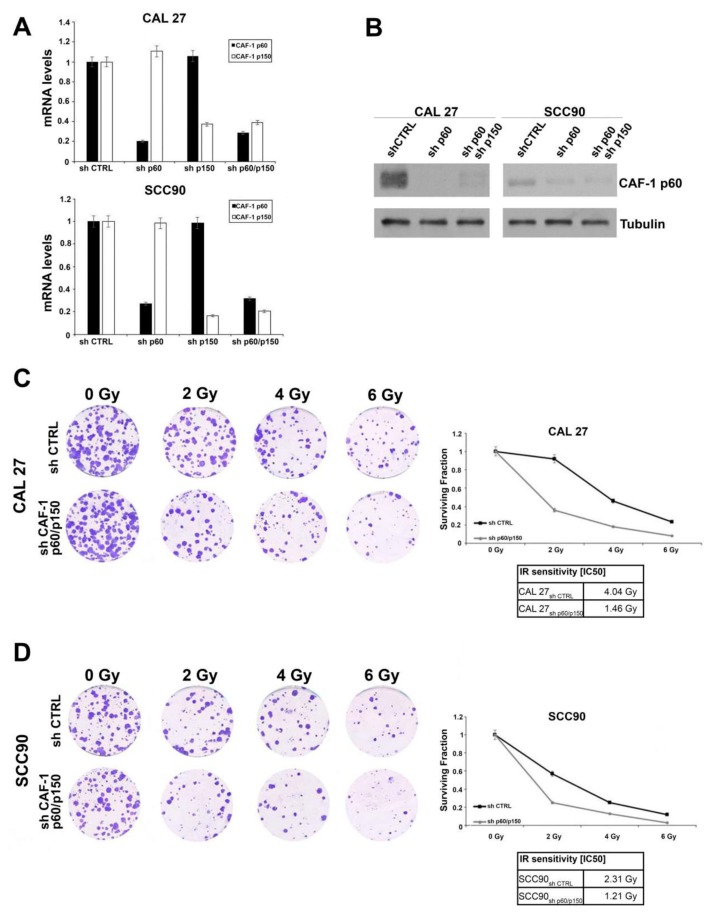
(**A**) Analysis of CAF-1 p60 and p150 relative mRNA expression by quantitative RT-PCR in human OSCC (CAL 27 HPV-negative) and OPSCC (SCC90 HPV-positive) cell lines, following stable trasnfection of the sh-CAF-1 p60, sh-CAF-1 p150 or both the sh-CAF1 p60/p150 vectors. (**B**) Immunoblot analysis of the CAF-1 p60 protein levels in OSCC- and OPSCC- derived cell lines. Anti-tubulin is shown as loading control. (**C**,**D**) (left) Clonogenic assay of CAL 27 and SCC90 cells after 0, 2, 4, and 6 Gy irradiation. Only colonies consisting of at least 30 cells were counted. (right) Clonogenic survival curve of CAL 27 and SCC90 cells. Error bars indicate the standard error mean. The sensitivity to ionizing radiation is expressed as IC_50_ (the value of radiation able to inhibit the cell growth of 50%).

The CAL27 cells (OSCC, HPV-negative) and SCC90 cells (OPSCC, HPV-positive) were silenced for the large subunit p150 and for the small subunit p60 of CAF-1, alone or in combination. The efficiency of silencing was evaluated by quantitative Real-Time-PCR (qRT-PCR), using a specific set of primers for both subunits of CAF-1. The analysis of the transcripts demonstrated low mRNA levels for the two proteins upon single or combined silencing (Figure 3A). The CAF1/p60 protein levels were evaluated in the p60 and in the p60 and p150 stably silenced cells, with Western blot (Figure 3B). The detection of the silencing efficacy of the p150 CAF-1 subunit was not possible at the protein level due to the failure of anti-p150 antibody. The standard therapy for non-metastatic head and neck cancer is based on ionizing radiation and/or surgery. However, metastatic lesions are treated with chemo- and radiotherapy, with a poor prognosis. In recent years, the possibility to characterize the presence and activation of the HPV virus in head and neck tumours has allowed the stratification of such tumours by the onset site with the prediction of response to therapies. The HPV-positive tumours showed high sensitivity to radiation, whereas the HPV-negative tumours resulted in a more resistant phenotype.

In search of the mechanisms responsible for the different behaviours of the HPV-positive and HPV-negative tumours, we investigated the role of CAF-1 subunits in determining the sensitivity to IR in head and neck cancer. As a model, we utilized two cell lines of head and neck tumours, which were confirmed for the HPV status (CAL 27 HPV-negative and SCC90 HPV-positive).

To evaluate whether the silencing of the CAF-1 subunits p60 and p150 (as single silencing or in combination) could affect the sensitivity to IR, we treated the CAL27 and SCC90 with range doses of IR (0, 2, 4, and 6 Gy). After ten days from irradiation, we analysed the rate of survival by a Colony Forming Assay (CFA) (Figure 3C,D). A significant reduction in the doses of radiations able to decrease the survival rate in 50% of the cells was observed. These effects were more evident in the shp60/shp150-silenced cells (IC_50_ CAL 27: shp60/p150: 1.46 Gy vs. shCTRL: 4.04 Gy; IC_50_ SCC90: shp60/p150: 1.2 Gy vs. shCTRL: 2.3 Gy) (Figure 3E).

The silencing of both the subunits of CAF-1 in our HPV-negative and HPV-positive cellular models resulted in an increased sensitivity to ionizing radiations. 

### 2.3. The Silencing of the Subunits of CAF-1 Leads to Defect in DNA Repair Mediated by Homologous Recombination and Sensitizes OSCC and OPSCC Cells to PARP-Inhibitors

**Figure 4:**
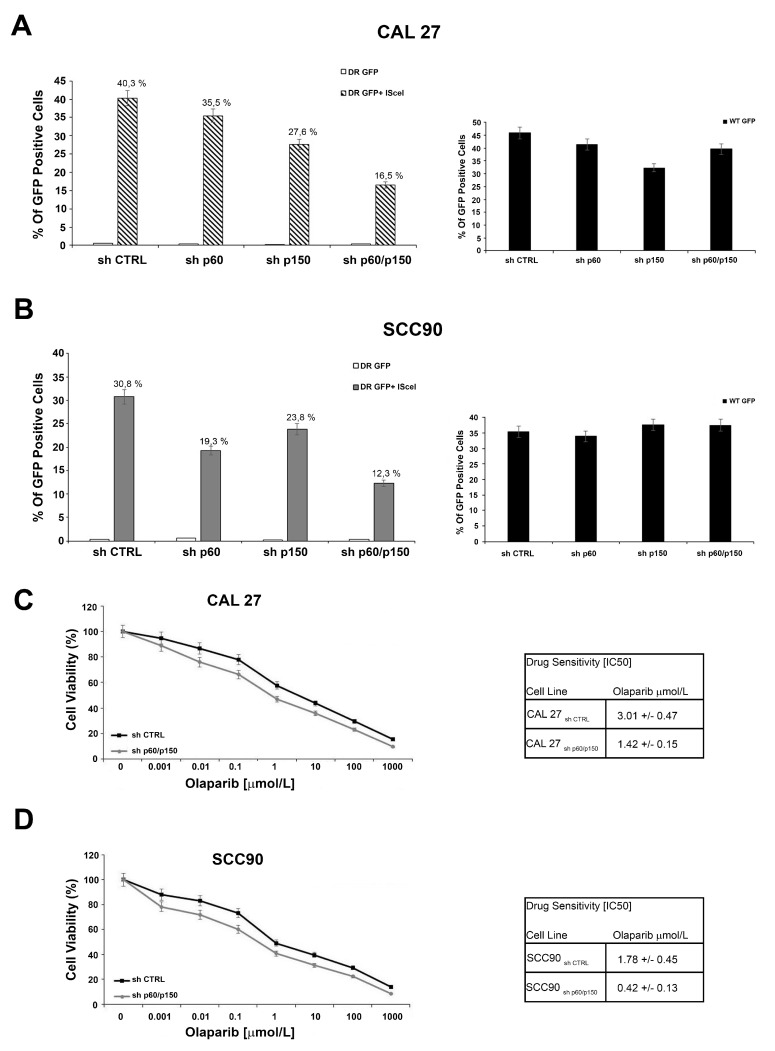
(**A**,**B**) Silencing of p60 and p150 suppresses homologous recombination. CAL 27 and SCC90 cells were transfected with DR-GFP alone, as control, or together with I-SceI. The percentage of GFP positive cells, compared to controls, is plotted on histograms representative of three independent experiments. Error bars indicate the standard error mean. (**C**,**D**) Silencing of p60 and p150 increases the sensitivity to Olaparib independently HPV-status. Survival fractions of CAL 27 and SCC90 cells treated with Olaparib, at the indicated doses, in presence (sh CTRL) or absence of CAF-1 (sh p60/p150) for 144 h. The sensitivity to the Olaparib was determined by the modified MTT assay (MTS), Cell Titer 96 AQueous One Solution assay, and expressed as IC_50_, i.e., 50% of the inhibitory concentration. The values are expressed as mean  ±  the standard deviation.

CAF-1 proteins have been reported to be involved in DNA repair mechanisms [28,29]. To investigate the proficiency of Homologous Recombination DNA damage repair in CAF-1 silenced cells, CAL27 and SCC90 cells were transfected with the DR-GFP reporter plasmid alone, as a control, or together with the I-SceI plasmid, able to induce DSBs. The ability to repair the DSBs by HR was measured by flow cytometry, and the frequency of HR is reported as a percentage of GFP positive cells. The silencing of both subunits determined a significant decrease of the GFP positive cells, compared to the control, suggesting that reduction of CAF-1 levels affected the DNA repair by HR. These results were obtained in both cellular models (Figure 4A,B). The HR proficiency was also assessed in cells stably silenced with shp60 or shp150 (as single silencing), obtaining similar results. Then, as defects in DNA repair by HR are reported to increase the sensitivity to inhibitors of the Poly(ADP-ribose) polymerase 1 and 2 (PARP1 and PARP2) [30,31], we evaluated the effects of the CAF-1/p150 and p60 subunits in modulating the CAL27 and SCC90 cells’ sensitivity to the PARP-inhibitor Olaparib. We treated cells with different concentrations of Olaparib and quantified the cytotoxic effect by a cell viability assay. Silencing of both the subunits of CAF-1 significantly increased the sensitivity to Olaparib (IC_50_ CAL27 shp60/p150CAF-1: 1.42 μM vs. shCTRL: 3.01 μM; IC_50_ SCC90 shp60/p150CAF-1: 0.42 μM vs. shCTRL: 1.78 μM) (Figure 4C,D). The impact of p150/p60 silencing and Olaparib effects on DNA double strand breaks with gamma H2AX staining was also assessed in the same experimental conditions (Figure S1).

### 2.4. Treatment with PARP-Inhibitors Increases the Radiosensitivity in HPV-Negative Head and Neck Cancer Cells

**Figure 5:**
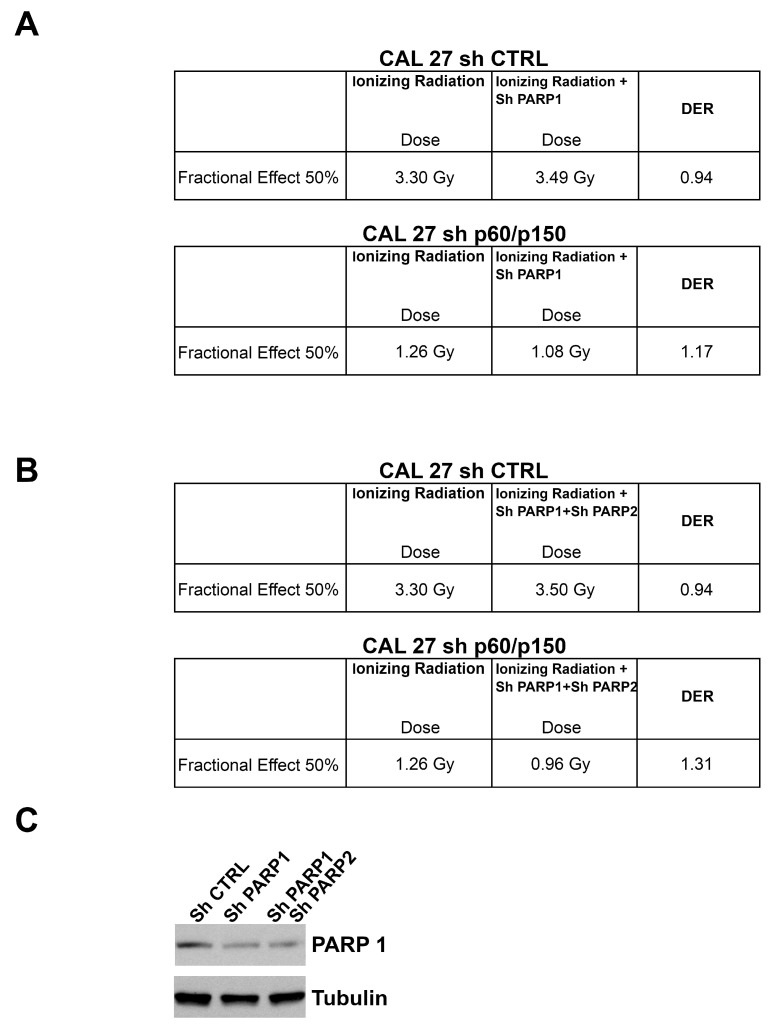
(**A**,**B**) The Dose Enhancement Ratio (DER) at 50% of the effect refers to the ratio between the dose with radiation alone and the dose with radiation + shPARP1 alone or in combination with shPARP2 for the same biological effect. If the DER is greater than one, then the silencing of PARP1/2 is functioning as a radiosensitizer. If the DER is less than one, the PARP silencing acts as a radioprotector. In the table are shown DER values at 50% effect for CAL 27 (sh CTRL vs. sh p60/p150). (**C**) The PARP1 depletion was assessed by Western blot. Tubulin is shown as loading control.

We finally analysed whether the silencing of CAF-1 might induce a radiosensitization effect in stably silenced cells upon PARP inhibitor treatment. The CAL 27 and SCC90 silenced cells and controls were plated and exposed to arange IR doses (0, 1, 2, 3, and 4 Gy), upon treatment with Olaparib at a fixed dose [0.1 μM]. Interestingly, by transfecting control shRNAs (shCTRL), shPARP1 and shPARP2, alone or together, in CAL27 cells (wild type or p150/p60 knockdown) and in SCC90 cells (wild type or p150/p60 knockdown), we yielded a radiosensibilizing effect similar to with PARP-inhibitors, as shown in Figure 5.

**Figure 6:**
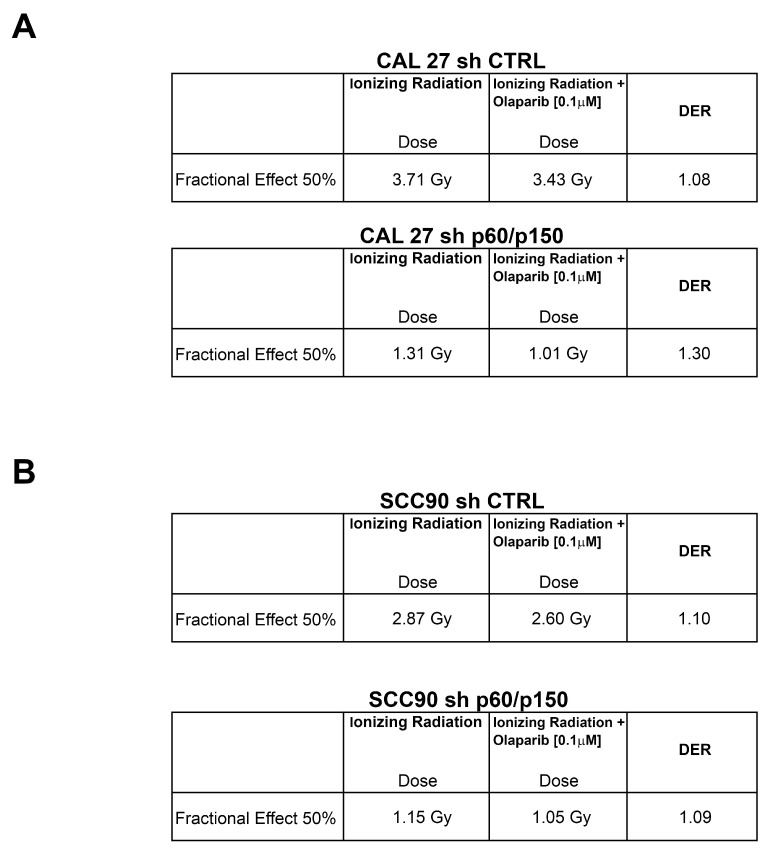
(**A**,**B**) The Dose Enhancement Ratio (DER) at 50% of the effect refers to the ratio between the dose with radiation alone and the dose with radiation + drug for the same biological effect. If the DER is greater than one, then the addition of the drug is functioning as a radiosensitizer. If the DER is less than one, the drug acts as a radioprotector. In the table are shown DER values at 50% effect for CAL 27 (sh CTRL vs. sh p60/p150) and SCC90 (sh CTRL vs. sh p60/p150).

We calculated cell survival by viability assay at 144 h (Figure 5). The results of the combined treatment were extremely relevant, as analysed by the Dose Enhancement Ratio (DER) calculated at 50% of cells’ survival [32]. The HPV-negative CAL27 cells showed a DER > 1 after cells exposure to different doses of ionizing radiation (0–4 Gy) in the presence of Olaparib [0.1 μM], which suggested a radiosensitization effect upon CAF-1 subunits depletion. However, the HPV-positive SCC90 cells, showed a moderate radiosensitization effect following a combined treatment, either in CAF-1 subunits depleted cells or in control cells (Figure 6). These data seem very promising as the SF2Gy, a standard measure of cells sensitivity to IR highly utilized in the field of radiobiology, indicates a great radiosensibilization effect upon CAF1 subunits silencing (Table S3).

## 3. Discussion 

Despite the recent advances in early diagnosis and surgical management of HNSCC, the outcome of these tumours has not substantially changed during the last decades, and the prognosis, for each HNSCC patient, remains linked to the tumour’s stage at presentation. The standard of care for these patients consists of radical surgery, complemented by radiotherapy, chemotherapy and, recently, immunotherapy in the case of advanced disease. The five-year survival rate for HNSCC patients remains poor, with up to 50% of mortality rates [1]. The most relevant limits of radio- and chemotherapy are represented by systemic and/or local toxicity, and by the frequent radio/chemoresistance of these tumours with particular attention to OSCC [33].

The limited information available on the molecular determinants of the biology of HNSCC indicates the urgent need to identify new prognostic markers and/or molecular targets for personalized therapeutic strategy [34]. 

Oral/oropharyngeal carcinogenesis mostly depends on environmental factors, such as tobacco and alcohol abuse, particularly in the oral cavity, and persistent infection from HR-HPV in a rising percentage of oropharyngeal cancers. Thus far, only a few reliable prognostic markers have been reported, alone or in combination, for OSCC and OPSCC [35,36,37,38,39]. 

The HPV status (persistent infection by HR-HPV, mostly HPV-16), only for oropharynx SCC, has been indicated, by the new TNM classification (AJCC 8th edition) as a significant marker of a more favourable biological behaviour of tumours, as has been shown by several studies in the last decade [40,41,42,43,44]. In HPV-negative tumours, mutations of TP53 and amplification of the Epidermal Growth Factor Receptor (EGFR) have been reported and used for prognostic and intervention indications with dismal results [45,46,47,48,49]. 

Chromatin remodelling proteins play an important role in genome maintenance processes, including DNA repair and replication, also involved in the development and progression of several human malignant tumours. Chromatin Assembly Factor 1 (CAF-1) is a “histone chaperone”, which delivers newly synthesized H3/H4 dimers to the replicative fork during the DNA synthesis phase (S) of the cell cycle [50]. CAF-1 is a heterotrimeric protein, of which p48 subunit cooperates with the Retinoblastoma protein (Rb) [51] and p150 and p60 subunits are involved in DNA repair and replication processes. Noteworthy, a leading role of p60 in sustaining the proliferative activity of cells in different malignancies has been described in the last two decades [14,52,53,54].

Recently, the presence of defects in genes involved in DNA damage repair by homologous recombination (HR) opened the way to the use of PARP inhibitors, alone or in association with genotoxic agents, such as ionizing radiation, in some malignant tumours [55]. The inhibition of PARP enzymes as an anticancer strategy has been established because of the biological concept of synthetic lethality, for which two genomic events, individually not lethal, become lethal when occurring together. When PARP enzymes are pharmacologically inhibited, the DNA single-strand breaks cannot be repaired and eventually progress to toxic double-strand breaks (DSBs), which result in being lethal in cells that lack HR repair capacity or have lost DNA repair genes [56,57,58]. In head and neck cancer cell lines characterized by a different ability to repair the DNA double-strand breaks through HR, the effectiveness of combined irradiation and PARP-inhibitor treatment in HR-deficient cells has been evaluated [59]. Very interestingly, it has been demonstrated that HPV positive head and neck tumours, carrying defects in DNA damage repair by HR, result sensitive to ionizing radiation [60]. 

Since HNSCC rarely carry mutations in DNA repair genes, there is considerable interest in finding alternative determinants of PARPi sensitivity. Our in vitro study, carried out by depleting CAF-1/p60 and p150 subunits (singularly or together), showed impairment of DSBs DNA repair and increased sensitivity to PARP inhibitor Olaparib in both cell lines used. This suggests that the presence of HPV, in the OPSCC cell line, did not modify the sensitivity of tumour cells with respect to the HPV-negative OSCC cell line. As a further step, we evaluated whether the treatment with Olaparib could modulate the response to ionizing radiation in the CAF-1 depleted cell lines. By combining ionizing radiation and PARP inhibitors drug, we observed an increased radio-sensitivity. Of particular interest, this effect resulted more evident in the OSCC CAL27 cells depleted of the CAF-1 p60/p150 subunits, as indicated by the DER that resulted greater than 1 (DER > 1). These data suggest that the combined treatment with ionizing radiation and PARP inhibitors can lead to the increase of the radio-sensitivity of OSCC, characteristically resistant to standard radio-treatment regimens.

All of the tumour samples included in the present study were re-examined and re-staged according to the eighth edition of the cancer staging manual of AJCC, and a “stage migration” was observed, as reported in Table S4 [61].

Classification through cluster analysis of immunohistochemical data allowed us to stratify the patients’ outcome in different subgroups: (i) a p60^low^/p150^low^ subgroup, mainly characterized by a good prognosis in both OSCC and OPSCC; (ii) a p60^high^/p150^low^ group which showed, mainly in OSCC, the worst outcome prediction; and (iii) a p60^high^/p150^high^ subgroup with an intermediate behaviour. These results confirm our preliminary data, obtained in the tongue tumour samples, of a prognostic role of the CAF-1/p60 subunit, which correlated with poor outcome and the importance of p60/p150 dual assessment as a prognostic determinant in OSCC tumours [20,24,25]. Interestingly, the survival data frequency distribution analysis, upon crossing the clusters by the overall survival results and the age groups, gave an extremely significant result in the mid-age population. Nevertheless, the Cox multivariate analysis, performed including age and stage variables together with p60/p150 clusters, revealed that p60/p150 staining is an independent prognostic factor (Figure S2). 

Our data let us envisage novel potential therapeutic approaches for OSCC by blocking the p60 protein, hampering the CAF-1 complex function and inducing HR defects that would sensitize tumour cells to Olaparib and/or in association with ionizing radiation.

We did not obtain significant results in OPSCC cohort, probably due to the small number of HPV+ in our series of cases. Interestingly, the only tumour with poor outcome among the HPV+ OPSCC showed the more aggressive immunophenotype (p60^high^/p150^low^) observed in OSCC.

However, this finding is not sufficient, at present, to propose the evaluation of CAF-1/p60 and p150 protein expression for prognostic and predictive stratification also of this tumours’ subset. For this reason, the evaluation of CAF-1 proteins’ expression needs to be evaluated on a larger, multi-institutional case study before we can lead to a definitive conclusion. This study is currently in progress.

In conclusion, our data confirm the reliability of CAF-1/p60 subunit as prognostic marker for OSCC, indicating in addition that the combined evaluation of p60 and p150 subunit may be of particular utility in stratifying the different prognostic classes of OSCC. 

In addition, we showed that CAF-1/p60 and p150 subunits are involved in HR-DDR, thus indicating the chance to induce a radiosensitizing synthetic lethality mechanism by treating tumour cells pharmacologically inhibited for CAF-1/p60 and p150 with PARP inhibitors, in OSCC patients in the worse prognostic group, in the direction of a truly personalized therapy.

## 4. Materials and Methods

### 4.1. Cell Lines and Drugs

Experiments were carried out using two human head and neck squamous cell lines, the CAL27 (Oral Squamous Cell Carcinoma, OSCC, HPV-negative) and SCC90 cells (Oropharyngeal Squamous Cell Carcinoma, OPSCC, HPV-positive). CAL27 cells were obtained by the “American Type Culture Collection” (ATCC) and SCC090 cell line was obtained by Leibniz Institute DSMZ-German Collection of Microorganisms and Cell Cultures, Germany.

Cell lines were cultured in the DMEM plus 10% of fetal bovine serum (Gibco, Paisley, UK).), L-Glutamine (2mM) and 100 U/mL of penicillin-streptomycin (Gibco, Paisley, UK) in 5% of CO_2_ at 37 °C. Olaparib (AZD2281) was provided by SelleckChem (Houston, TX, USA).

### 4.2. Real Time PCR

PCR reactions were performed on RNA isolated from cell lines using the RNeasy Mini Kit (Qiagen, Hilden, Germany) and reverse-transcribed using MuLVRT (Invitrogen, Carlsbad, CA, USA). The qRT-PCR analysis was performed with Syber Green (Agilent, Santa Clara, CA, USA). Primer sequences are reported in Table S5. The relative expression levels were calculated by the 2-ΔΔCT method.

### 4.3. Western Blotting and Antibodies

Western blotting was performed as described (Figures S3 and S4) [62,63]. Immunoblotting experiments were carried out according to standard procedures and visualized using the ECL chemiluminescence system (Amersham/Pharmacia Biotech, Little Chalfont, UK). Anti-CHAF1B (HPA021679) and anti-Tubulin were provided by SIGMA-Aldrich, Inc. Secondary antibodies were from Biorad (Hercules, CA, USA).

### 4.4. Plasmids and Transfection

MISSION shRNA (pLKO.1) NM_005441 (CHAF1B MISSION shRNA Plasmid DNA, cod: TRCN0000074278, TRCN0000074279, and TRCN0000074281] and NM_005483 [CHAF1A MISSION shRNA Plasmid DNA, cod: TRCN0000074273 and TRCN00000234596) and shRNA of control (sh CTRL) were utilized for stable transfection and were provided by Sigma-Aldrich, Inc. (St. Louis, Missouri, USA) For transfection, the FuGENE 6 Transfection Reagent was provided by Promega Italia S.r.l. (Milano, Italy). 

### 4.5. Sensitivity Test and Design for Drug Combination

Antiproliferative activity was determined by a modified 3-(4,5-dimethylthiazole-2-yl)-2-5- diphenyltetrazolium bromide assay, CellTiter 96 AQueous One Solution Assay (Promega, Milano, Italy), calculated as 50% inhibitory concentration (IC_50_) values, according to the manufacturer instructions.

Briefly, cells were plated in quintuplicate in 96-well plates at a density of 1000 cells per well, and continuously exposed to each drug for 144 h. Each assay was performed in quintuplicate and IC_50_ values were expressed as mean ± standard deviation. 

The results of the combined treatment were expressed as a DER, calculated at 50% of survival. DER is a measure of how many folds each radiation dose may be reduced when administered in combination with a drug to obtain a given effect, in comparison to the same dose of radiation when given alone. A value of DER greater than one (DER > 1) indicates that a fixed dose of the drug used, in association with a range doses of IR was able to act as radiosensitizer, while a value of DER lower than one (DER < 1) indicates that a fixed dose of the drug used, in association with a range of IR doses, works as a radioprotector [32,64].

### 4.6. Colony Forming Assay

One-hundred-millimeter-cubed dishes of proliferating cells were exposed to 0, 2, 4, and 6 Gy of IR. After cells counting, a pre-defined number of viable cells were plated in 6-well plates, in triplicate. To receive a sufficient colony count, a different number of cells were plated for each dose of irradiation (0 Gy, 200 cells; 2 Gy, 500 cells; 4 Gy, 600 cells; and 6 Gy, 800 cells) [65]. After 10 days of incubation, prior to counting colonies, cells were stained with 0.5% Crystal Violet (10 min at room temperature). A population of at least 30 cells was scored as one survivable colony and considered for the count. The colonies’ counting was performed at the optic microscope and through the open source software ImageJ-NIH [66]. The relative colony formation (surviving fraction) was expressed as the number of colonies per treatment level versus colonies that appeared in the untreated control. (mean colony counts ± standard errors are reported).

### 4.7. TMA and IHC

One hundred fifty-four HNSCC FFPE tumour samples (112 OSCC and 42 OPSCC, of which 8 HPV-positive), from surgical resections, were used to build tissue micro-arrays (TMAs). The study was performed according to the guidelines of the Institutional Ethic Committee, which, in agreement with the Italian law, with reference to the topics of the current research and according to the Declaration of Helsinki require, for studies based only on retrospective analyses on routine archival FFPE-tissue, a written informed consent from the living patient, following the indication of Italian DLgs No. 196/03 (Codex on Privacy), as modified by UE 2016/679 law of European parliament and Commission at the time of surgery.

The HPV positivity was confirmed through p16 immunostaining and HPV genotyping by INNO Lipa [67]. Seven TMAs were built selecting the most representative areas from each selected paraffin block, at least in duplicate. Three-millimeter tissue cores were punched from morphologically representative tissue areas of each donor block and placed into one recipient paraffin block (3 cm × 2.5 cm) using a semi-automated tissue arrayer (Galileo TMA, Milan, Italy). To ensure a sufficient representation of the tissue composition of individual cancer cases, in view of the known frequency of tumour heterogeneity in H&N SCC, we built TMAs taking 3-mm cores in at least duplicate or triplicate (in the case of bigger tumours). We already challenged our TMA protocol with whole slide assessment of tissue biomarkers expression (please see reference mentioned below) finding a high degree of agreement between the CAF-1/p60 assessment on TMAs and on routine tissue sections [54]. One section of each TMA (4 μm) was stained with hematoxylin and eosin (H&E) to check the adequacy of cores. The immunohistochemical staining with anti-CAF-1/p60 and CAF-1/p150 (ab8133 and ab126625 obtained from AbCam, Cambridge, UK) [68] were evaluated semi-quantitatively as the percentage of positive cells (with either nuclear or cytoplasmic localization) among the total number of tumour cells and classified as LOW staining (including 0 (<5% di cellule positive); and + (5% to <15%) scores) and HIGH staining (including ++ (15% to <30%); +++ (>30%) scores). All immunoassayed TMA glass slides were digitalized with an Aperio AT2 digital pathology slide scanner (Leica Biosystems Nussloch GmbH, Heidelberger, Germany) and visualized with QuPath image software analysis [69,70,71]. 

### 4.8. Statistical Analysis

Statistical analysis was performed using SPSS software (IBM Corp. Released 2013. IBM SPSS Statistics for Windows, Version 22.0. Armonk, NY, USA). Tissue biomarker expression scores’ correlation with the overall survival variable was performed by Fisher exact test. A K-mean cluster analysis was performed to sort relatively homogeneous groups of cases based on selected characteristics (CAF-1/p60 and CAF-1/p150 IHC expression). Survival analysis was performed by test Kaplan–Meier survival curves’ differences by log-rank test. *p*-value was considered significant at *p* = 0.05.

### 4.9. HR Reporter Assay

The DR-GFP reporter and pCAGGS-I-SceI plasmids were used to verify the functionality of DNA repair mechanism by HR. The DR-GFP reporter plasmid, based on a construct developed by M. Jasin [72], consists of two mutated and GFP negative expression cassettes (GFP-I, GFP-II). In Cassette I, there is a unique cutting site recognized by the restriction enzyme I-SceI. In cells transfected with the DR-GFP and the I-SceI plasmids, the expression of I-SceI induces a double-stranded break (DSB) in Cassette I. This damage can be repaired through HR, using the GFP gene present as a template in Cassette II and thus restoring the expression of GFP (Figure 1). The ability of cells to repair double-stranded damage by HR was measured by flow cytometry reporting the percentage of positive GFP cells as a measure of HR proficiency.

## 5. Conclusions

The immunohistochemical analysis of CAF-1/p60 and CAF-1/p150 tissue expression in OSCC revealed a significant prognostic value of the combined expression of both tissue biomarkers, while no significant prognostic value was demonstrated in OPSCC. In HPV-positive and HPV-negative cell lines, the p60/150 CAF-1 subunits depletion impaired the proficiency of Homologous Recombination (HR) DNA damage repair, inducing sensitivity to the PARP-inhibitors drugs able to sensitize both the cell lines to ionizing radiations. These results indicate that, regardless of the prognostic meaning of the expression of the two markers, the pharmacological depletion of CAF-1 complex’s function, combined to PARP-inhibitors and/or radiotherapy, could represent a valid therapeutic strategy for squamous cell carcinomas of head and neck region, by the mean of a synthetic lethality mechanism.

## Figures and Tables

**Table 1:** Descriptive statistics of the studied population. NOP, non-oropharyngeal tumours; OP, oropharyngeal tumours. HPV positivity (p16 IHC) is only reported for oropharyngeal squamous cell carcinomas.

Study Population		Frequency	%
Stage	I	15	9.7
	II	30	19.5
	III	18	11.7
	IVA	56	36.4
	IVB	12	7.8
	Missing	23	14.9
Sex	F	72	46.8
	M	82	53.2
Age	Mean	63.8	
	Median	64	
	Std Dev	13.1	
	Range	57	
	Min	33	
	Max	90	
p60 score	HIGH	107	69.5
	LOW	47	30.5
p150 score	HIGH	26	16.9
	LOW	128	83.1
p60/p150 combined score	p60HIGH/p150HIGH	26	16.9
	p60HIGH/p150LOW	81	52.6
	p60LOW/p150LOW	47	30.5
Tumour site	NOP	112	72.7
	OP	42	27.3
HPV (p16)	NEG	34	81
	POS	8	19
F-up (months)	Mean	32.92	
	Median	24	
	Mode	12	
	Range	156	
	Min	1	
	Max	157	
Tot		154	100

## Supplementary Materials

The following are available online at https://www.mdpi.com/2072-6694/11/10/1582/s1, Table S1: Contingency table showing frequency distribution of IHC expression data crossed with overall survival, Table S2 (A): The table shows the distribution of the three clusters frequency counts stratifying according to stage, age class and outcome, Table S2 (B): Demographic table showing the distribution of cases count frequency grouping by age class and outcome and stratifying by CAF p60/p150 clusters. The distribution was statistically significant in mid age class at Chi-squared test of significance (*p* = 0.002), Table S3: Type one (α) and type two (β) DNA damage in Cal27 Sh CTRL and CAL27 Sh p60/p150, using linear quadratic radiobiological model, Table S4: 34 out of 154 samples were upstaged in the staging score, 17/34 (50%) of the upstaged patients died at follow-up. The table shows the cunt of upstaged samples at the restaging according to the 8th edition of AJCC TNM manual. A&W = alive and well. DOD = death of disease, Table S5: Oligi sequence of the utilized primers, Figure S1: The percentage of g-H2AX positive nuclei at 1h and 4h from irradiation are in A and B. Error bars represent standard error mean. Results are representative of at least two independent experiments, Figure S2: p60/p150 tissue exprssion is an independent prognosti factor in a Cox multivariate analysis including also age and stage variables (global significance of the model *p* = 0,008, HR of p60^high^/p150^low^ equal to 1789.
