# Supplementary material for: CAF-1 Subunits Levels Suggest Combined Treatments with PARP-Inhibitors and Ionizing Radiation in Advanced HNSCC
Source: Cancers (Basel). 2019 Oct 17;11(10):1582. doi: 10.3390/cancers11101582 (PMC6827109; doi:10.3390/cancers11101582)

Supplementary Materials: CAF-1 Subunits Levels Suggest Combined Treatments with PARP-Inhibitors and Ionizing Radiation in Advanced HNSCC

**Table S1.** Contingency table showing frequency distribution of IHC expression data crossed with overall survival.

| **Contingency table** | | | | | | |
| --- | --- | --- | --- | --- | --- | --- |
| Tumor | P150 |  | | OS | | Tot. |
|  |  |  |  | Alive | Dead |  |
| OSCC  P = 0.013 | HIGH | P60 | HIGH | 5 | 8 | 13 |
|  |  | Tot. | | 5 | 8 | 13 |
|  | LOW | P60 | HIGH | 18 | 45 | 63 |
|  |  |  | LOW | 20 | 16 | 36 |
|  |  | Tot. | | 38 | 61 | 99 |
|  | Tot. | P60 | HIGH | 23 | 53 | 76 |
|  |  |  | LOW | 20 | 16 | 36 |
|  |  | Tot. | | 43 | 69 | 112 |
| OPSCC  P = 0.485 | HIGH | P60 | HIGH | 8 | 5 | 13 |
|  |  | Tot. | | 8 | 5 | 13 |
|  | LOW | P60 | HIGH | 10 | 8 | 18 |
|  |  |  | LOW | 8 | 3 | 11 |
|  |  | Tot. | | 18 | 11 | 29 |
|  | Tot. | P60 | HIGH | 18 | 13 | 31 |
|  |  |  | LOW | 8 | 3 | 11 |
|  |  | Tot. | | 26 | 16 | 42 |
| OSCC+  OPSCC  P = 0.022 | HIGH | P60 | HIGH | 13 | 13 | 26 |
|  |  | Tot. | | 13 | 13 | 26 |
|  | LOW | P60 | HIGH | 28 | 53 | 81 |
|  |  |  | LOW | 28 | 19 | 47 |
|  |  | Tot. | | 56 | 72 | 128 |
|  | Tot. | P60 | HIGH | 41 | 66 | 107 |
|  |  |  | LOW | 28 | 19 | 47 |
|  |  | Tot. | | 69 | 85 | 154 |

**Table S2.** (**A)** The table shows the distribution of the three clusters frequency counts stratifying according to stage, age class and outcome.

|  | | **Clusters** | | |
| --- | --- | --- | --- | --- |
|  |  | **60H150H** | **60H150L** | **60L150L** |
|  |  | **Counting** | **Counting** | **Counting** |
| Stage8th | I | 0 | 6 | 9 |
|  | II | 9 | 12 | 9 |
|  | III | 3 | 12 | 3 |
|  | IVA | 8 | 30 | 18 |
|  | IVB | 0 | 8 | 4 |
|  | NA | 6 | 13 | 4 |
| Age classes | med | 13 | 25 | 22 |
|  | old | 12 | 55 | 22 |
|  | young | 1 | 1 | 3 |
| OS | 0 | 13 | 28 | 28 |
|  | 1 | 13 | 53 | 19 |

**Table S2.** (**B)** Demographic table showing the distribution of cases count frequency grouping by age class and outcome and stratifying by CAF p60/p150 clusters. The distribution was statistically significant in mid age class at Chi-squared test of significance (*p* = 0.002).

|  | | **Age classes** | | | | |
| --- | --- | --- | --- | --- | --- | --- |
|  |  | **med** | | **old** | | **young** |
|  |  | **OS** | | **OS** | | **OS** |
|  |  | 0 | 1 | 0 | 1 | 0 |
| Clusters | 60H150H | 8 | 5 | 4 | 8 | 1 |
|  | 60H150L | 6 | 19 | 21 | 34 | 1 |
|  | 60L150L | 16 | 6 | 9 | 13 | 3 |

**Table S3.** Type one (α) and type two (β) DNA damage in Cal27 Sh CTRL and CAL27 Sh p60/p150, using linear quadratic radiobiological model.

|  | **Radiation** | | |
| --- | --- | --- | --- |
|  | CAL27 Sh CTRL | CAL27 Shp60/p150 | Shp60/p150 / Sh CTRL Ratio |
| α | 5,69 | 12,42 | 2,18 |
| β | 2,26 | 0,43 | 0,19 |
| α/β | 2,52 | 25,54 | 10,13 |

**Table S4.** 34 out of 154 samples were upstaged in the staging score, 17/34 (50%) of the upstaged patients died at follow-up. The table shows the cunt of upstaged samples at the restaging according to the 8^th^ edition of AJCC TNM manual. A&W= alive and well. DOD= death of disease.

| Upstaged Cases | | | |
| --- | --- | --- | --- |
|  |  | OS | |
| From 7h | to 8th | A&W | DOD |
| I | II | 2 | 3 |
| I | III | 1 | - |
| II | III | 5 | 4 |
| II | IVA | 0 | 1 |
| III | IVA | 3 | 2 |
| IVA | IVB | 5 | 7 |

**Table S5.** Oligi sequence of the utilized primers.

| **Target** | **Forward primer (5’-3’)** | **Reverse primer (3’-5’)** |
| --- | --- | --- |
| CHAF- 1 p60 | CGGGTCCCTCCAGCATTTT | GACAGCATCATCTCCTCCCG |
| CHAF- 1 p150 | TCCCATCTCGCTGAAGAGGA | CATACGTCACCCCTGCTCTC |
| GAPDH | GTCTTCACCACCATGGAGAA | ATCCACAGTCTTCTGGGTGG |


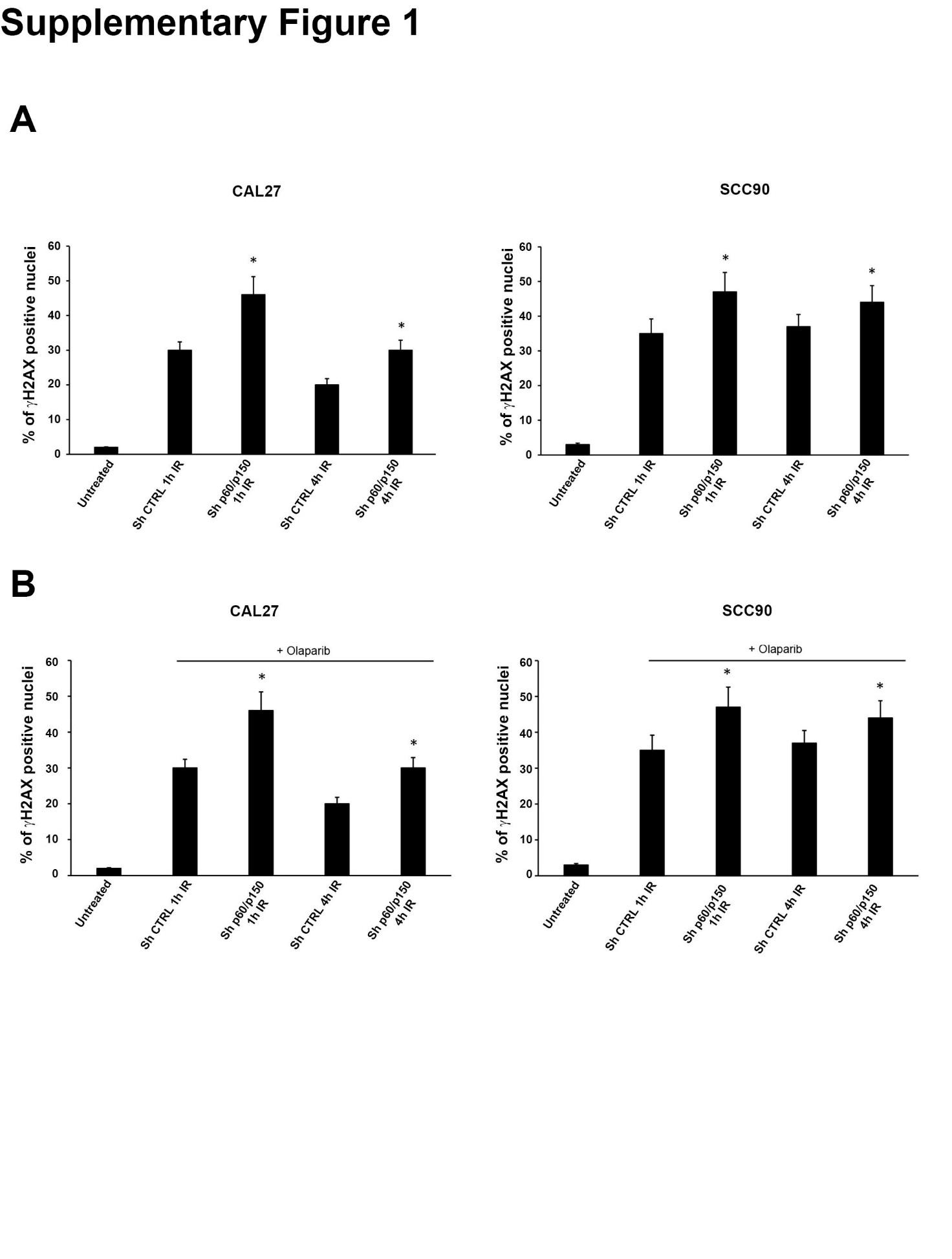


**Figure S1.** The percentage of g-H2AX positive nuclei at 1h and 4h from irradiation are in A and B. Error bars represent standard error mean. Results are representative of at least two independent experiments.

| **Test Omnibus of Model Coeffcient** | | | | | | | | | |
| --- | --- | --- | --- | --- | --- | --- | --- | --- | --- |
| Log likelihood-2 | Global (score) | | | Modified from previous phase | | | Modified from previous block | | |
|  | Chi-squared | gl | Sign. | Chi-squared | gl | Sign. | Chi-squared | gl | Sign. |
| 664,999 | 18,914 | 7 | ,008 | 19,282 | 7 | ,007 | 19,282 | 7 | ,007 |

| **Variable in the Equation** | | | | | | |
| --- | --- | --- | --- | --- | --- | --- |
|  | **B** | **SE** | **Wald** | **gl** | **Sign.** | Exp(B) |
| newcomb |  |  | 4,935 | 2 | ,085 |  |
| newcomb(1) | -,455 | ,345 | 1,737 | 1 | ,187 | ,634 |
| newcomb(2) | ,581 | ,285 | 4,172 | 1 | ,041 | 1,789 |
| AGE | ,015 | ,010 | 2,307 | 1 | ,129 | 1,015 |
| Stage8th |  |  | 10,116 | 4 | ,039 |  |
| Stage8th(1) | -,415 | ,548 | ,572 | 1 | ,449 | ,661 |
| Stage8th(2) | -,378 | ,472 | ,640 | 1 | ,424 | ,685 |
| Stage8th(3) | -,734 | ,527 | 1,943 | 1 | ,163 | ,480 |
| Stage8th(4) | ,251 | ,427 | ,345 | 1 | ,557 | 1,285 |

| **Covariate mean and model values** | | | | |
| --- | --- | --- | --- | --- |
|  | Mean | Modello | | |
|  |  | 1 | 2 | 3 |
| newcomb(1) | -,181 | ,667 | -,333 | -,333 |
| newcomb(2) | ,005 | ,333 | ,333 | -,667 |
| AGE | 64,168 | 64,168 | 64,168 | 64,168 |
| Stage8th(1) | ,115 | ,115 | ,115 | ,115 |
| Stage8th(2) | ,229 | ,229 | ,229 | ,229 |
| Stage8th(3) | ,137 | ,137 | ,137 | ,137 |
| Stage8th(4) | ,427 | ,427 | ,427 | ,427 |


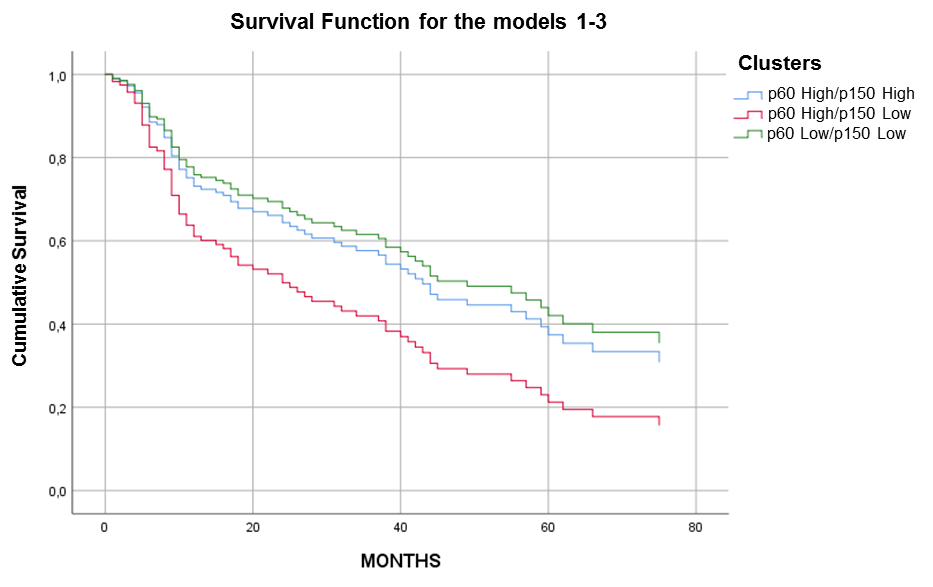


**Figure S2.** p60/p150 tissue exprssion is an independent prognosti factor in a Cox multivariate analysis including also age and stage variables (global significance of the model p =0,008, HR of p60^high^/p150^low^ equal to 1,789.


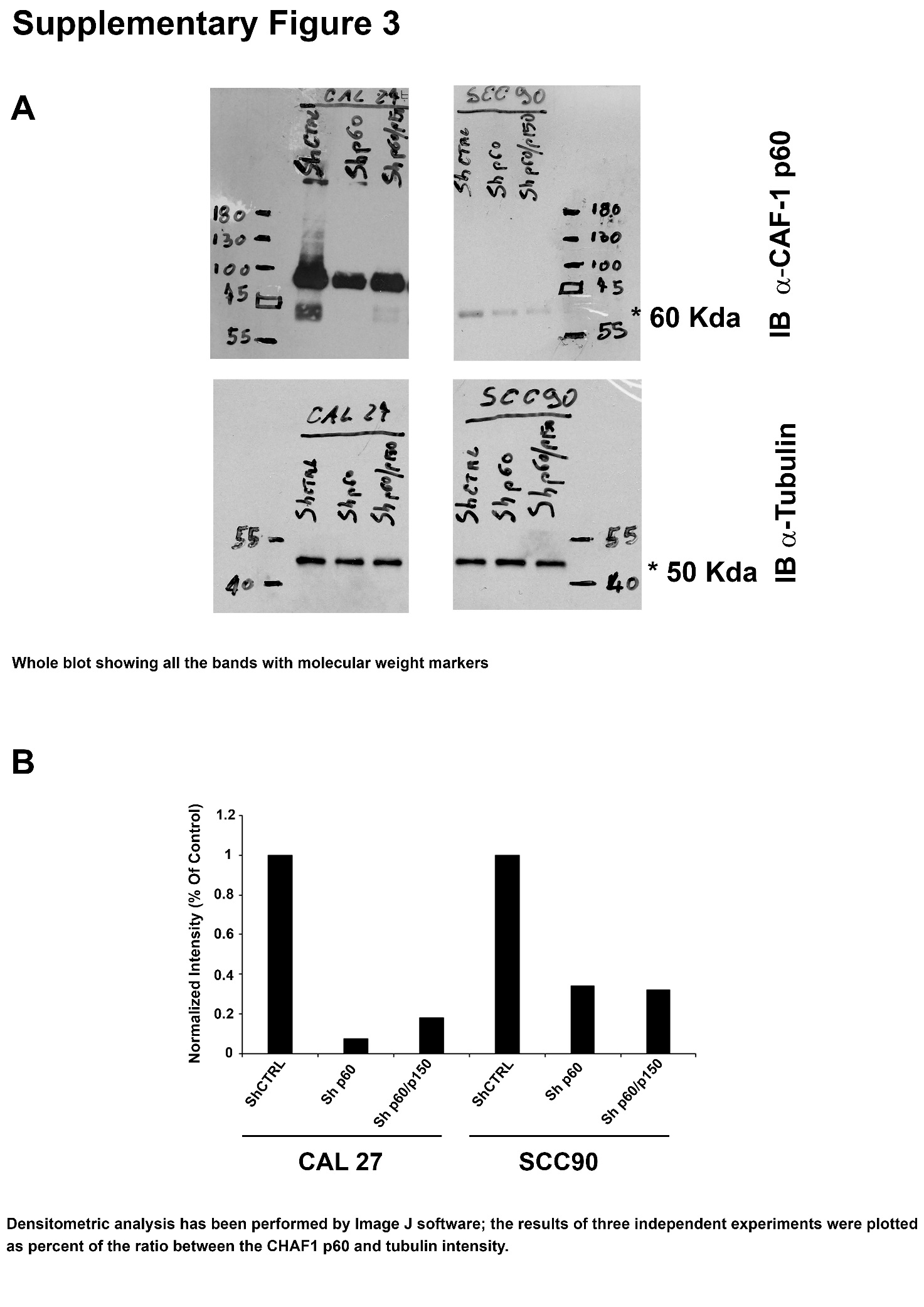


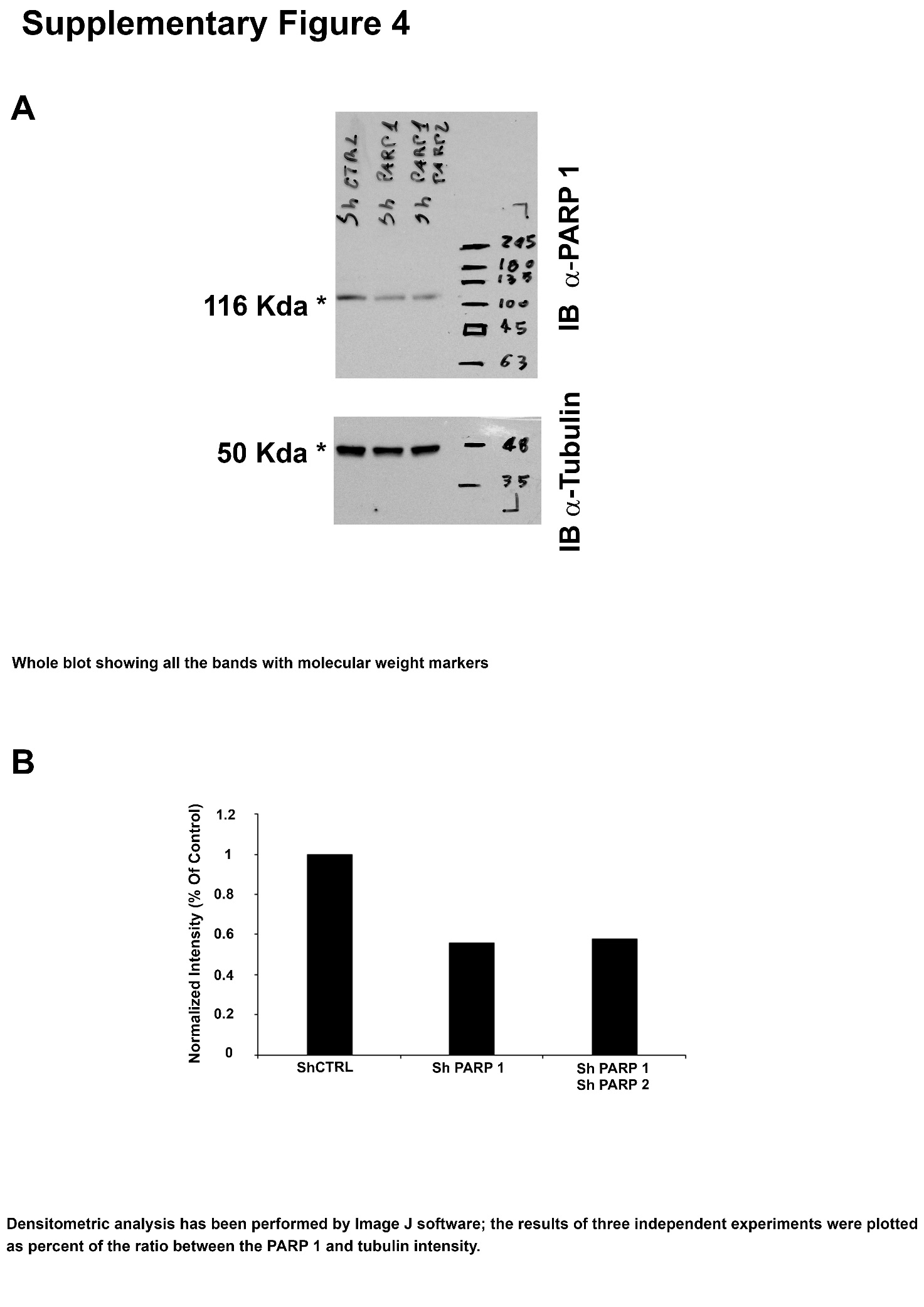

Supplement: Supplementary file 1 [file cancers-11-01582-s001.docx]
